# Identification and analysis of a cell communication prognostic signature for oral squamous cell carcinoma at bulk and single‐cell levels

**DOI:** 10.1111/jcmm.70166

**Published:** 2024-11-24

**Authors:** Xingwei Zhang, Fan Yang, Chen Dong, Baojun Li, Shuo Zhang, Xiaohui Jiao, Dong Chen

**Affiliations:** ^1^ Department of Oral and Maxillofacial Surgery The First Affiliate Hospital of Harbin Medical University Harbin China; ^2^ Department of Stomatology The First Affiliated Hospital of Heilongjiang University of Chinese Medicine Harbin China; ^3^ Department of Beauty and Plastic Surgery Heilongjiang Provincial Hospital Harbin China; ^4^ Department of Head and Neck Surgery Harbin Medical University Cancer Hospital Harbin China

**Keywords:** immune microenvironment, prognostic model, oral squamous cell carcinoma

## Abstract

Head and neck squamous cancer (HNSC) is a heterogenous malignant tumour disease with poor prognosis and has become the current major public health concern worldwide. Oral squamous cell carcinoma (OSCC) is the majority of HNSC. It is still in lack of comprehensive tumour immune microenvironment analysis and prognostic model development for OSCC's clinic practice. Single‐cell sequencing data analysis was conducted to identify immune cell subtypes and illustrate cell–cell interaction status in OSCC via R package ‘Seurat’, ‘Harmony’, ‘elldex’ and ‘CellChat’. Base on the bulk sequencing data, WGCNA analysis was employed to identify the CD8^+^ T cell related gene module. XGBoost was used to construct the gene prognostic model for OSCC. Validation sets and immunotherapy data sets were analysed to further evaluate the model's effectiveness and immunotherapy responsiveness predicting potential. siRNA was used to down regulate FCRL4 expression. Real‐time PCR and Western blot were used to validate target gene expression. The effects of FCRL4 on OSCC cells were detected by wound healing, Trans well and clone formation assays. Communication between epithelial cells and tissue stem cells may be the potential key regulators for OSCC progression. By integrating single‐cell sequencing data analysis and bulk sequencing data analysis, we constructed a novel immune‐related gene prognostic model. The model can effectively predict the prognosis and immunotherapy responsiveness of OSCC patients. In addition, the effects of FCRL4 on OSCC cells were validated. We comprehensively interpreted the immune microenvironment pattern of OSCC based on the single‐cell sequencing data and bulk sequencing data analysis. A robust immune feature‐based prognostic model was developed for the precise treatment and prognosis evaluation of OSCC.

## 1 INTRODUCTION

As one of the major public health concerns worldwide, head and neck squamous cancer (HNSC) has more than 930,000 victims and causes over 460,000 deaths around the world each year.^1^ Among the HNSC, more than 90% of them are oral squamous cell carcinoma (OSCC).^2^, ^3^, ^4^ Current therapy strategies for OSCC include surgery, radiotherapy, and chemotherapy.^5^, ^6^, ^7^ Although these therapy options significantly elevated patient prognosis due to the therapy resistance, about 30% of the patients suffer from tumour recurrence, and the five‐year survival rate is only 50%–60%.^8^, ^9^ Immune checkpoint inhibitors (ICIs), involving PD‐1/PD‐L1 inhibitors, provided the opportunity for the treatment of OSCC, which is resistant to traditional therapy strategies. However, there is a non‐negligible rate of patients who performed limited responsiveness to ICI.^10^ A better understanding of immune microenvironment is helpful in predicting immune escape.^11^ Tumour immune microenvironment and infiltrating immune cells are the deciding characteristics for the response of ICI.^12^, ^13^, ^14^, ^15^ Therefore, characterizing tumour microenvironment and evaluating immune infiltration levels are important to identify the candidates for immunotherapy of OSCC.

The frequent development of high‐throughput sequencing technology allowed us to further illustrate the immune microenvironment of cancer. Long et al. identified two HNSC subtypes with distinct immune microenvironment patterns based on features of TNF family proteins and analysed the regulating role of TNF family proteins in HNSC.^16^ However, bulk sequencing can only provide an imprecise illustration of tumour microenvironment patterns. The analysis of cellular heterogeneity in tumour tissues is limited. By separating and obtaining transcripts of each cell, single‐cell RNA sequencing (scRNA‐seq) technology could focus on intratumor heterogeneity and help researchers identify immune cell subsets in the immune microenvironment.^17^ Some clustering methods have been developed for unifying cell‐type recognition and subtype identification, such as unifies cell type recognition and subtype identification (UCRSI), which improved the visualization of large‐scale cell clustering.^18^ A serious of studies depicted the characterizes of OSCC based on scRNA‐seq. Le Meitour et al. uncovered the immune checkpoint heterogeneity of OSCC based on scRNA‐seq data, their identified three subgroups of patients with distinct immune phenotypes.^19^ Wu et al. revealed the role of cancer‐associated fibroblasts in OSCC using scRNA‐seq. they established risk model was correlated with immune‐related cells and immune‐related genes.^20^ By employing scRNA‐seq, Puram et al. identified the signatures of partial epithelial‐to‐mesenchymal transition (p‐EMT) and also demonstrated it as an independent prognostic of cancer metastatic activities.^21^ Thus, the integration of bulk sequencing and scRNA‐seq data can help us further explore the immune microenvironment heterogeneity of OSCC and the related precise treatment strategy.

In this study, we aimed to construct a cell–cell communication‐related prognostic model to identify OSCC signatures. At first, the immune cell subsets in OSCC were identified based on scRNA‐seq and we illustrated the cell–cell communication pattern in the immune microenvironment. Then, the immune‐related gene modules were identified via WGCNA based on the bulk sequencing data. By integrating the results of analysis based on the bulk sequencing and scRNA‐seq data, we identified a series of tumour immunity signatures. Next, we constructed a prognostic model using XGboost method.^22^ The model performed well in both training sets and validation sets. Immunotherapy cohorts‐based analysis demonstrated that our model has great potential in the identification of OSCC immunotherapy candidates. In addition, the effects of FCRL4 on OSCC cells were validated in OSCC cells. The integrated results indicated that our prognostic model may be applied in the clinic for precise treatment of OSCC.

## 2 METHODS AND MATERIALS

### 2.1 Bulk data collection

Genomic information, as well as clinical data of HNSC samples from Cancer Genome Altas (TCGA), were retrieved from Genomic Data Commons (GDC, https://portal.gdc.cancer.gov/).^23^ A total of 304 oral squamous cell carcinoma (OSCC) samples with survival information were extracted from HNSC samples, including 18 alveolar ridge samples, 22 buccal mucosa samples, 60 floors of mouth samples, seven hard palate samples, 72 oral cavity samples, and 125 oral tongue samples. Gene expression microarray of 97 OSCC samples (GSE41613) Gene Expression Omnibus (GEO) (http://www.ncbi.nlm.nih.gov/geo) was employed to obtain related clinical data.^24^

IMvigor210 is a single‐arm phase II research to investigate anti‐PD‐L1 drug (atezolizumab) within metastatic urothelial carcinoma (mUCC) patients.^25^ IMvigor210 trial expression and clinical information were acquired through ‘IMvigor210CoreBiologies’ R package.

### 2.2 Single‐cell RNA sequencing (scRNA‐seq) data collection and preparation

ScRNA‐seq of six OSCC samples and eight HNSC samples were obtained from GEO database (GSE172577 and GSE164690). R softwares ‘Seurat’ and ‘Harmony’ were employed for quality controlling, batch effect elimination, and integrating data within 14 distinct samples (GSM5258385, GSM5258386, GSM5258387, GSM5258388, GSM5258389, GSM5258390, GSM5017023, GSM5017032, GSM5017035, GSM5017041, GSM5017044, GSM5017047, GSM5017050 and GSM5017062).^26^ For cell‐quality filtering, we used Seurat's Normalize Data function to standardize the raw data. Some functions such as ‘FindVariableFeatures’ and ‘ScaleData’ were also used. And cells that had either lower than 200 expressed genes or higher than 6000 expressed genes were removed. Furthermore, we discarded cells with mitochondria UMI rates of more than 10%. We searched highly variable genes using a variance‐stabilizing transformation method. We selected the highest 2000 most variable genes for principal component analysis (PCA). Then, we ran ‘Harmony’ on the first 50 PCs, and performed clustering with a resolution of 1, obtaining the corrected PC embeddings finally. On the same distance metric, cells were represented utilizing a 2‐dimensional *t*‐distributed stochastic neighbour embedding (t‐SNE). The ‘SingleR’ package was applied to annotate cell types using HumanPrimaryCellAtlasData provide by ‘celldex’ package.

The cell–cell communications between cell types were investigated utilizing R software ‘CellChat’. Researchers concentrated on the human dataset In CellChat as well as identified over‐expressed ligands or receptors by ‘identifyOverExpressedGenes’ and ‘identifyOverExpressedInteractions’ functions. Then, we mapped gene expression data into PPI network by ‘projectData’ function. The functions ‘computeCommunProb’ and ‘filterCommunication’ were employed to estimate communication probabilities and infer cellular communications network. ‘computeCommunProbPathway’ and ‘aggregateNet’ algorithms were implemented to infer cell–cell communication among every cell type at signalling pathway level.

### 2.3 Identification of immune module by weighted gene co‐expression network analysis (WGCNA)

Cell‐type identification by estimating relative subsets of RNA transcripts (CIBERSORT) is a technique which employs the input matrix of a gene expression file to precisely calculate the relative proportions of different cell subsets in tissues.^27^ We inferred immune cells infiltration in TCGA OSCC samples utilizing CIBERSORT analysis. WGCNA is an unsupervised categorization and data‐reducing approach.^28^ Co‐expression network was established depending on gene expression profile in TCGA utilizing ‘WGCNA’ tool in the R program to identify a module which is related to immune infiltration in CIBERSORT but not affected by clinical features (including sex, age, outcome, TNM stage, and tumour stage and tumour grade). Clustering of genes and drawing of heat map to demonstrate the correlation between modules and phenotype. Blue module was eligibility for choosing.

### 2.4 Functional analysis for integrated gene set

Epithelial cells and tissue stem cells frequently had cell communications according to scRNA‐seq analysis. The top 5 differentially expressed genes (DEGs) in epithelial cell clusters and tissue stem cell clusters were identified. Together with genes in blue module, 688 genes were collected.

We conducted a pathway and process enrichment study for 688 genes using a variety of ontology resources, including KEGG Pathway, GO Biological Processes, Reactome Gene Sets and Canonical Pathways, using the Metascape web‐based software (https://metascape.org/gp/index.html).^29^ The Metascape analysis was conducted with default adjustment (*p* < 0.01, minimal count of 3, as well as enrichment factor >1.5). For more studying of the association among the terms, a network plot was developed using a subset of selected enriched terms, in which terms with a similarity >0.30 are connected by edges.

### 2.5 Identification of clinical outcome‐related genes by XGBoost machine learning model

Extreme Gradient Boosting (XGBoost), is a scalable, distributed gradient‐boosted decision tree machine learning algorithm.^30^ XGBoost algorithm is based on gradient enhanced decision tree and adopts second‐order Taylor expansion to calculate the loss function, which has good performance in computation speed and prediction accuracy. The XGBoost algorithm has excellent advantages in handling missing values, preventing overfitting and reducing run time.^31^ An XGBoost gradient‐boosted tree model with was used to identify clinical outcome‐related genes in integrated 688 genes in TCGA OSCC dataset by R package ‘xgboost’, with parameter of max_depth = 2, eta = 0.3, silent = 1, objective = ‘binary:logistic’. ‘Gain’ is a metric defined by XGBoost and it also involves evaluation of the structure of the tree. A total of 15 genes with gain >0.015 were retained.

### 2.6 Construction of the prognostic model

Employing the ‘survival’ R software, a multivariate Cox regression model was developed dependent on optimized genes. Risk score was measured by expression of 15 genes and coefficients from a multivariate Cox regression model.

Calculation of risk score was done as the following:

Risk score = Σ(Expi × coefi).

### 2.7 Survival analysis

Patients in TCGA OSCC and IMvigor210 datasets were classified based on the mean risk score. Based on risk score tertiles, GSE41613 populations were categorized (the first two‐thirds as high risk and the last third as low risk).

Chi‐square test was performed to explore risk score associations with clinical features (T, N and M stage, age, sex and so on). Kaplan–Meier plots were utilized to display findings (Table 1).

**TABLE 1:** Baseline characteristics of patients in TCGA OTSCC cohort.

Characteristics	Whole cohort	High risk	Low risk	*p*
TCGA cohort	(*n* = 304)	(*n* = 152)	(*n* = 152)	
Gender				0.3916
Male	205 (67.43%)	106 (69.74%)	99 (65.13%)	
Female	99 (32.57%)	46 (30.26%)	53 (34.87%)	
Age				0.5625
<65 years	173 (56.91%)	89 (58.55%)	84 (55.26%)	
> = 65 years	131 (43.09%)	63 (41.45%)	68 (44.74%)	
T‐stage				0.015
T1	99 (65.13%)	99 (65.13%)	99 (65.13%)	
T2	53 (34.87%)	53 (34.87%)	53 (34.87%)	
T3	99 (65.13%)	99 (65.13%)	99 (65.13%)	
T4	53 (34.87%)	53 (34.87%)	53 (34.87%)	
N‐stage				0.7742
N0	160 (52.63%)	81 (53.29%)	79 (51.97%)	
N1	54 (17.76%)	26 (17.11%)	28 (18.42%)	
N2	77 (25.33%)	39 (25.66%)	38 (25%)	
N3	1 (0.33%)	0 (0%)	1 (0.66%)	
M‐stage				0.9961
M0	287 (94.41%)	143 (94.08%)	144 (94.74%)	
M1	2 (0.66%)	1 (0.66%)	1 (0.66%)	
Stage				0.1612
I	11 (3.62%)	2 (1.32%)	9 (5.92%)	
II	73 (24.01%)	40 (26.32%)	33 (21.71%)	
III	61 (20.07%)	31 (20.39%)	30 (19.74%)	
IV	151 (49.67%)	75 (49.34%)	76 (50%)	
Grade				0.9345
G1	48 (15.79%)	24 (15.79%)	24 (15.79%)	
G2	190 (62.5%)	93 (61.18%)	97 (63.82%)	
G3	62 (20.39%)	32 (21.05%)	30 (19.74%)	

### 2.8 Cell culture and transfection

In the current research, CAL‐27 cell line was utilized and they were cultured in 25 cm^2^ cell culture flasks (Corning, NYC, USA) which were filled with Dulbecco's Modified Eagle Medium (DMEM) (Invitrogen, Waltham, USA) that has been supplemented with 15% fetal bovine serum (Invitrogen, Waltham, USA). These cells were maintained in a CO_2_‐enriched environment at a temperature of 37°C. For the gene knockdown experiments, x‐treme GENE siRNA (Invitrogen, Carlsbad, USA) were employed for 24 h. The siRNA sequences that were used are available in the Table S1.

### 2.9 Real‐time RT‐PCR assay

The extraction of total RNA from the cell lines was carried out using the Trizol reagent (Invitrogen, Waltham, USA) following the manufacturer's protocols. Subsequently, cDNA was produced utilizing a reverse transcription reagent kit (TAKARA, RR037A, Shiga, JAPAN). The quantification of gene expression was performed using the SYBR Green PCR Master Mix and detected through the utilization of Roche 480 systems. For mRNAs, GAPDH were respectively employed as internal controls. The 2^−ΔΔCt^ relative quantification method was utilized to demonstrate gene expression, with the primer details provided in Table S2.

### 2.10 Wound healing assay and colony formation assays

To perform the wound healing assay, cells were cultivated in 6‐well plates until they reached approximately 90% confluence. The cell monolayer was then wounded using sterile 100‐μL pipette tips. Afterward, the cells were rinsed three times with D‐Hanks to eliminate any detached cells and subsequently incubated in RPMI 1640 supplemented with 5% FBS for 48 h. For the colony formation assay, cells were likewise cultured in 6‐well plates for a period of 2–3 weeks. The resulting colonies were quantified by staining with 1% crystal violet.

### 2.11 Statistical analysis

A one‐sided Wilcoxon rank‐sum test was utilized to examine the difference among high and low‐risk clusters. For the biological experiments, comparation between two groups or among three groups were conducted using *t*‐test or one‐way ANOVA, respectively. All statistical analyses were conducted applying R package. It was regarded statistically significant if *p* < 0.05.

## 3 RESULTS

### 3.1 Establishment of cell atlas in oral squamous cell carcinoma

**FIGURE 1:**
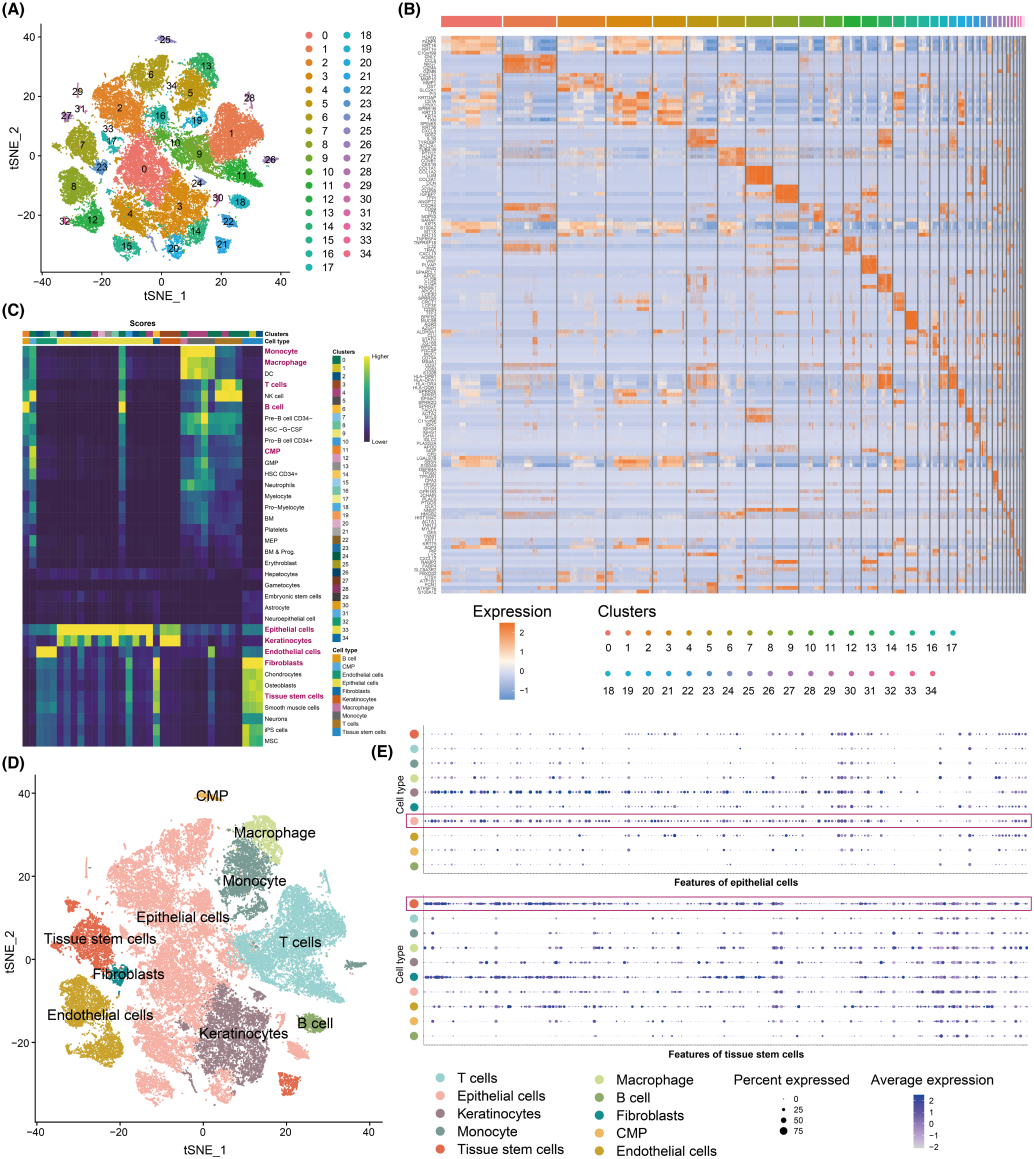
Single‐cell analysis for OSCC and HNSC samples. (A) Single‐cell profile's tSNE plot is coloured by clusters. (B) Heatmap depicting five significant DEGs for every cell cluster. (C) SingleR method was used for cell annotation. Heatmap of the assignment score for each cell (column) and label (row). (D) The tSNE plot showed the annotations of cell types by SingleR method. (E) The expression of cell markers of epithelial cells and tissue stem cells which provided in SingleR.

Analytical procedures in this research are demonstrated in Figure S1. We collected scRNA‐seq data for six OSCC and eight HNSC samples; Harmony method was used to remove the batch effect and integration of data (Figure S2). Then, 34 clusters were identified by seurat method (Figure 1A). Significant DEGs in every group were detected using the ‘FindAllMarkers’ function of the Seurat method, and the top five significant DEGs in every group were displayed using a heatmap (Figure 1B and Data S1). A total of cell types (epithelial cells, T cells, keratinocytes, monocyte, tissue stem cells, endothelial cells, macrophage, B cell, fibroblasts and CMP) were annotated by SingleR method (Figure 1C,D). The expression of cell markers of epithelial cell and tissue stem cell which provided in SingleR were shown in Figure 1E.

### 3.2 Analysis of cell communications in oral squamous cell carcinoma

**FIGURE 2:**
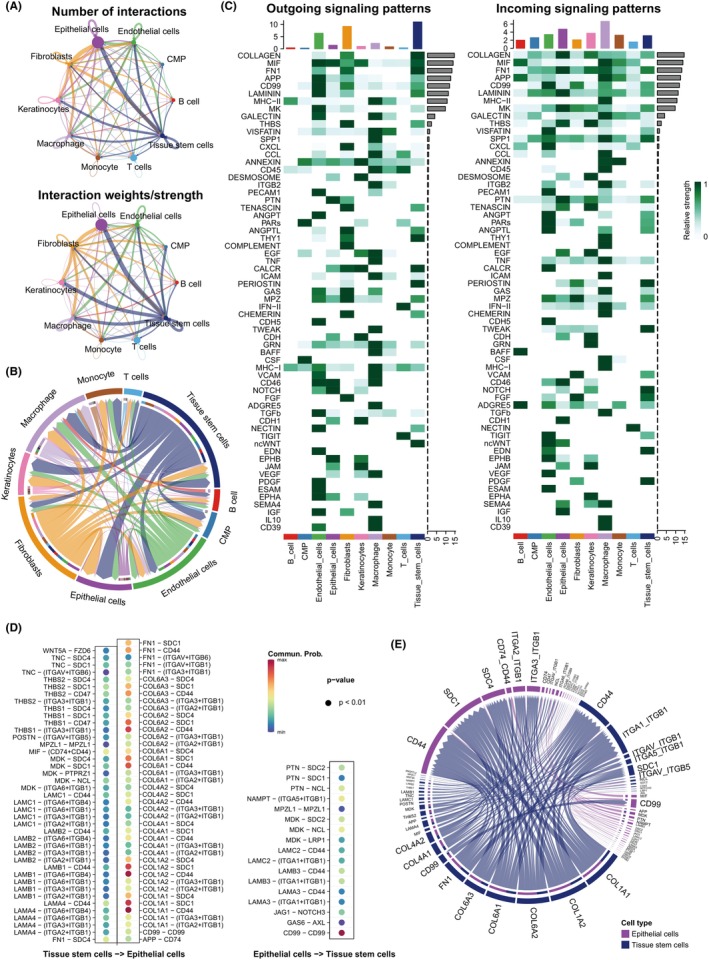
Identification of frequent cell communications among cell types. (A) Numbers and strength of interactions among cell types. (B) Chord diagram shows interactive pathways among the cell types. (C) Heatmap showing potential ingoing or outgoing signalling pathways between cell types. (D) Dot plot representing the potential pairs of ingoing or outgoing signalling pairs between epithelial cells and tissue stem cells. (E) Chord diagram indicates interactive signalling pairs between epithelial cells and tissue stem cells.

Cell–cell communication study was conducted for a better understanding of the integrating role of these types of cells in OSCC; these types interacting between each other are displayed in Figure 2A. Epithelial cells and tissue stem cells had frequent communications. Then, the interactive pathways were analysed (Figure 2B), and Potential ingoing and outgoing signals were examined (Figure 2C). The pathway communications have appeared more between tissue stem cells and epithelial cells. Tissue stem cells send more signal of COLLAGEN, MIF, and FN1 pathways and epithelial cells were received a lot of communications in COLLAGEN, FN1, LAMININ and MK pathways. Subsequently, the specific signal pairs between epithelial cells and tissue stem cells were investigated (Figure 2D,E). The results showed strong communications between epithelial cells and tissue stem cells through the COL1A1‐CD44, COL1A2‐CD44, COL1A1‐SDC1, COL1A2‐SDC1, etc.

### 3.3 Identification of immune‐related co‐expressed module

**FIGURE 3:**
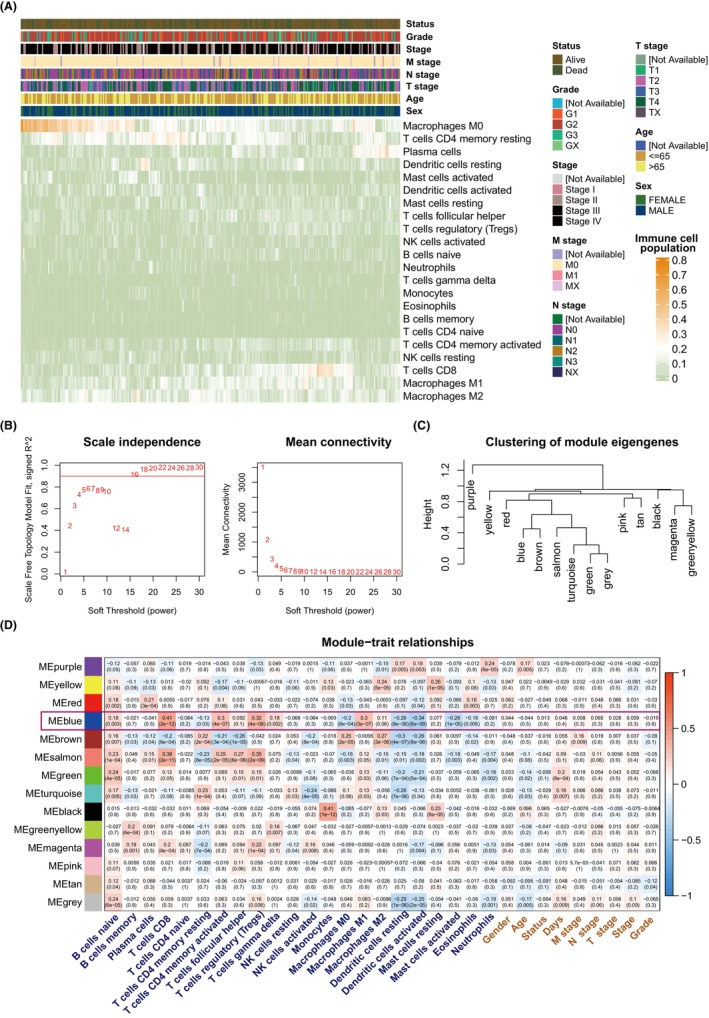
Identification of immune‐related co‐expressed module. (A) Abundance of 22 immune cells with the CIBERSORT approach in TCGA OSCC samples. (B) Index of scale‐free fit for soft‐threshold powers. (C) Relationships between WGCNA module clusters. (D) Association between immunological abundance, clinical characteristics and modules.

To characterize the immune microenvironment of OSCC, CIBERSORT was utilized for the calculation of proportion of 22 immune cell infiltration in bulk TCGA samples (Figure 3A).

For the identification of a group of genes whose expression patterns are associated with immune microenvironment, we construct co‐expressed network by WGCNA method based on the expression profile of TCGA OSCC. To assure median connectivity and high independence, we examined the modules power value. The power value was set at 16 as soft‐threshold variable for a scale‐free network (Figure 3B). A total of 14 modules were detected (Figure 3C, Figure S3). Module‐trait association study was employed to determine co‐expression modules which highly related to immune cell infiltration however unaffected by clinical characteristics. Figure 3D illustrates the modules association with phenotype. As per correlation analysis, blue module that contains 617 genes was detected due to association with immune cells, especially with CD8 T cells.

### 3.4 Determination of prognostic features and construction of a prognostic model

**FIGURE 4:**
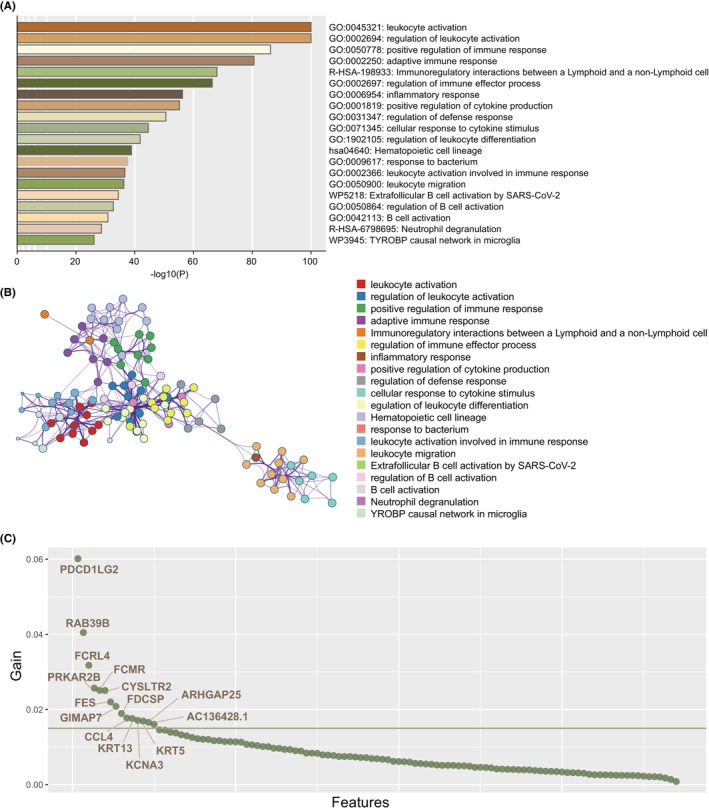
Functional analysis for integrated gene set and feature selection. (A) Pathway and process enrichment analysis have been performed on DEGs, which are defined among subtypes. The graphic presentation displayed top 20 enrichments with *p* < 0.01. (B) Enrichment terms similar to those larger than 0.3 are connected by edges. (C) Gain value distribution for genes evaluated by XGboost machine learning algorithm.

We integrated significant DEGs of epithelial cell and tissue stem cell clusters, and immune‐related blue module genes, and a total of 688 genes were gathered (Data S2). To investigate gene set potential biological behaviour, Pathway and process enrichment analysis were performed (Figure 4A,B). Multiple immune‐relevant pathways and processes involving ‘leukocyte activation,’ ‘regulation of leukocyte activation,’ ‘positive regulation of immune response,’ ‘adaptive immune response’, as well as ‘Immunoregulatory interactions among lymphoid and non‐lymphoid cells’, were significantly highly expressed in genes. (Data S3).

To further explore the genes that related to clinical outcome, XGboost machine learning algorithm was performed to select outcome‐related genes (Figure 4C). The detail parameters were displayed in Data S4. A total of 15 genes with gain >0.015 were retained (Data S5).

**FIGURE 5:**
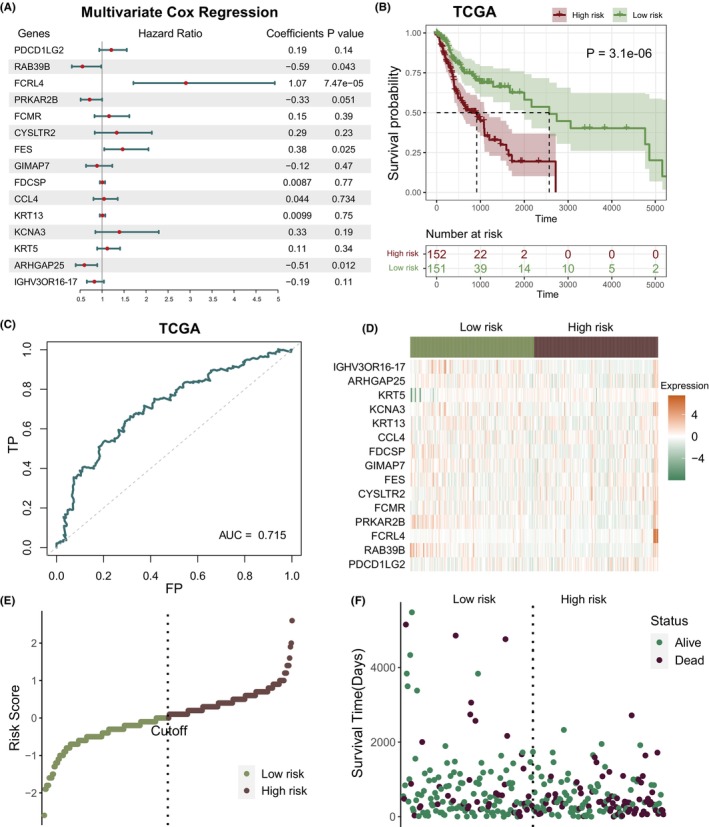
Construction and validation of the prognostic model. (A) A multivariate Cox proportional hazard model was built based on 15 genes. (B) Using log‐rank test, OS variance among high and low‐risk samples were evaluated in TCGA cohort (C) Prognostic model ROC curve in TCGA cohort. (D) Distribution of gene expression in prognostic model. (E) Distribution of risk score in TCGA cohort. (F) Distribution of survival time in TCGA cohort.

**FIGURE 6:**
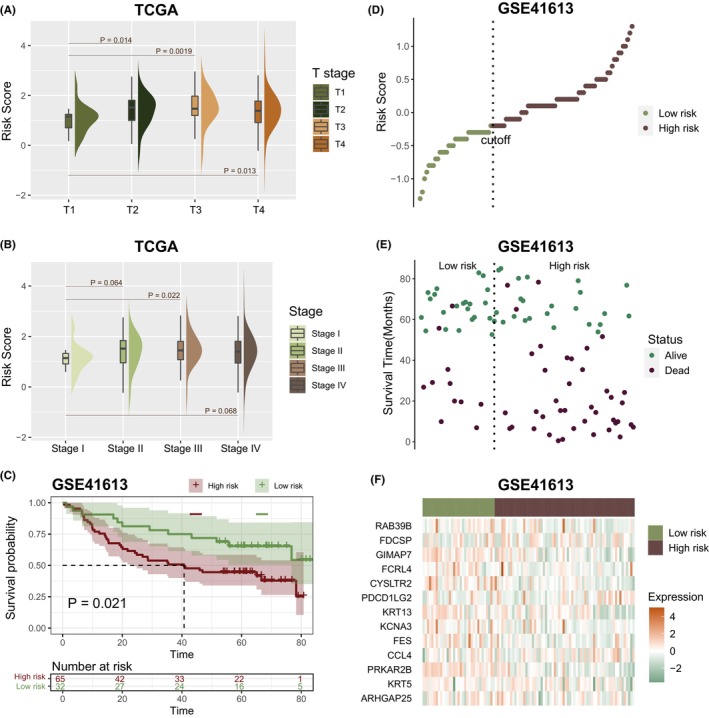
Validation of the prognostic model. (A) Risk score distribution among T‐stage groups in TCGA cohort. (B) Risk score distribution among clinical stages in TCGA cohort. (C) Log‐rank test was employed to detect OS variance among high and low‐risk samples within GSE41613 cohort. (D) Risk score distribution in GSE41613 cohort. (E) Distribution of survival time in GSE41613 cohort. (F) Distribution of gene expression in prognostic model.

To explore the prognostic value of optimized genes (PDCD1LG2, RAB39B, FCRL4, PRKAR2B, FCMR, CYSLTR2, FES, GIMAP7, FDCSP, CCL4, KRT13, KCNA3, KRT5, ARHGAP25 and IGHV3OR16–17), a mutivariate Cox proportional hazard model was built (Figure 5A). Then, depending on 15 genes expression and coefficients, a prediction model was constructed. In accordance with the abovementioned formula, risk score of every TCGA patient was determined. Participants were split to high and low risk categories utilizing mean as cutoff value. High‐risk category patients have a significant lower OS (Figure 5B, *p* = 3.1e‐06, log‐rank test). In accordance with area under curve (AUC) of receiver operating characteristic (ROC) curve, we identified that risk score provides an accurate prediction of death for 3 years (Figure 5C, AUC = 0.715). Figure 5D shows 15 genes expression in prognostic model. Figure 5E shows risk scores distributing throughout TCGA cohort. There were more alive patients in low risk cluster than in high risk cluster (Figure 5F). Besides, T2, T3 and T4 stages risk score were significantly greater than T1 (Figure 6A, *p* < 0.05, one‐sided Wilcoxon rank‐sum test). Stage II and Stage IV risk score were greater than Stage I, and Stage III risk score were significantly greater than Stage I (Figure 6B, one‐sided Wilcoxon rank‐sum test). Also, we validated our model in patients of different ages, genders and TMN stages and found that this model performed well across different patient types and could distinguish their survival times (Figure S4).

### 3.5 Validation of the prognostic model and prediction of immunotherapy

Next, we explored the predictive effect in independent validation databases. Risk scores in GSE41613 patients were calculated based on mutivariate Cox proportional hazard model. The tertiles of score were utilized to categorize participants into high and low‐risk categories. High‐risk category patients had a significantly poorer OS (Figure 6C, *p* = 0.021, log‐rank test). There were more alive patients in low‐risk category than in high‐risk category (Figure 6D). Risk score distribution in GSE41613 was displayed in Figure 6E. Expression of 13 genes in prognostic model is shown in Figure 6F

**FIGURE 7:**
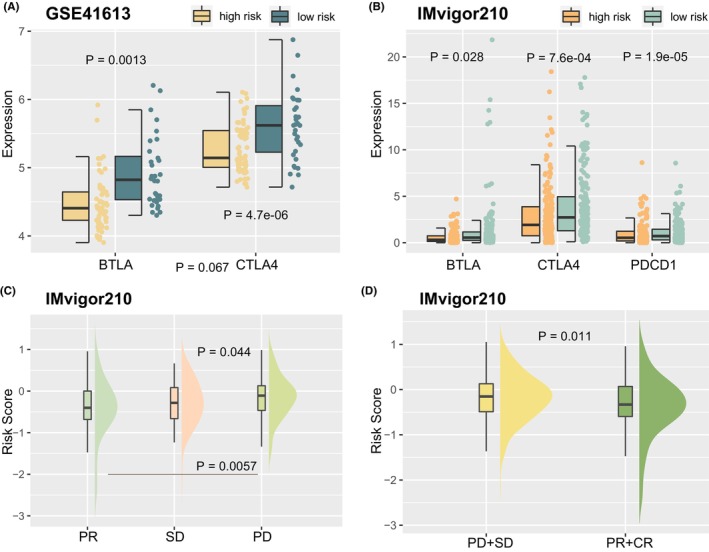
Prognosis power of model to immunotherapy response. (A) Expression of BTLA and CTLA4 between risk groups in GSE41613 cohort. (B) Expression of BTLA, CTLA4, and PDCD1 between risk groups in IMvigor210 cohort. (C, D) Risk score distribution among responsive groups in IMvigor210 cohort.

In addition, we explored risk model ability in predicting immunotherapy responses in patients. Expression of immune checkpoints BTLA and CTLA4 in low‐risk cluster were significantly greater than high risk cluster in GSE41613 cohort (Figure 7A, *p* < 0.05, one‐sided Wilcoxon rank‐sum test). Expression of BTLA, CTLA4, and PDCD1 in low risk category were significantly greater than high risk category in IMvigor210 cohort (Figure 7B, *p* < 0.05, one‐sided Wilcoxon rank‐sum test). Furthermore, risk score in PD (progressive disease) patients was significantly greater than in PR (partial response) and SD patients (stable disease) (Figure 7C, *p* < 0.05, one‐sided Wilcoxon rank‐sum test). Unresponsive patients (PD and SD) had significantly greater risk scores compared to responsive individuals (PR and CR [complete response]) (Figure 7D, *p* = 0.011, one‐sided Wilcoxon rank‐sum test).

### 3.6 Validation of the prognostic genes

**FIGURE 8:**
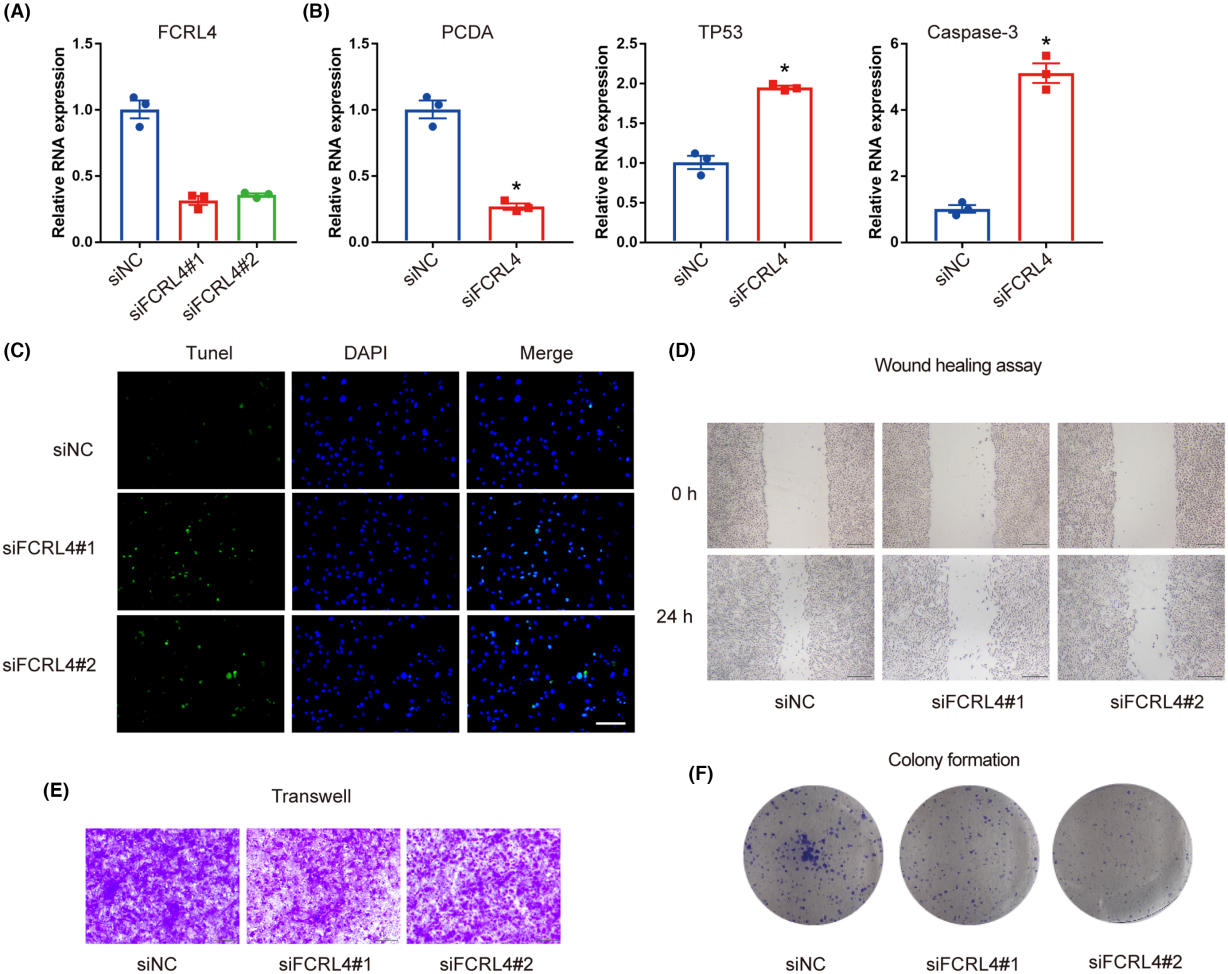
The effect of FCRL4 on OSCC cells. (A) The mRNA expression levels of FCRL4. (B) The mRNA expression levels of PCDA, TP53 and Caspase‐3. (C) The protein expression of TP53, cleaved‐Caspase‐3, PCDA and FCRL4. (E) The representative images of tunel experiments (bar: 100 μm).(D) The representative images of wound healing assay. (E) The representative images of Transwell assay. (F) The representative images of clone formation assay. *p < 0.05.

Based on the analysis results of bioinformatics, we then performed molecular biology experiments to validate the survival‐related gene function in cancer cell lines. Here we selected the top gene with highest Hazzard Ratio, FCRL4, to test the regulatory potential in key biological processes of cancer cells. We used two siRNAs to inhibit the expression of FCRL4 in CAL‐27 cells, results showed that siRNA no. 1 has stronger inhibiting ability, thus we used the siRNA no. 1 for further experiments (Figure 8A). Firstly, to investigate the regulative effects of FCRL4 in cell proliferation and apoptosis, we tested the relative expression of markers of proliferation (PCNA) and apoptosis (cleaved‐Caspase‐3) and the cancer suppressor TP53 via quantitative real‐time RT‐PCR. Results showed that inhibition of FCRL4 can suppress the expression of PCNA and promote the expression of TP53 and Caspase‐3 (Figure 8B). Furthermore, the results of Tunel experiments also demonstrated that knocking down FCRL4 could enhance the apoptotic activity of cancer cells (Figure 8C). These results indicated that FCRL4 is an important regulator in cancer cell proliferation and apoptosis. In addition, we also performed the wound healing assay, and colony formation assays to test the effects of FCRL4 in cancer cell invasion and metastasis (Figure 8D‐F). Results showed that inhibition of FCRL4 can repress cancer cell invasion and metastasis.

## 4 DISCUSSION

OSCC is a highly intratumor heterogeneous tumour type.^32^, ^33^ Integrated analysis of bulk sequencing data and scRNA‐seq data is urgently in demand to characterize immune microenvironment of OSCC.^34^ Based on scRNA‐seq data, Cornelius et al. identified an elastic differentiated fibroblast subtype in HNSC and revealed the role of cell communication between tumour cells, epithelial cells and stromal cells in tumour microenvironment progression.^35^ Wang et al. analysed the role of tumour immune microenvironment's CD8^+^ T cells, M1 macrophages and CCDC43 in cancer metastasis and prognosis.^36^ However, the research integrating the bulk sequencing data and scRNA‐seq data to further analyse tumour immune microenvironment of OSCC is still lacking.

Facing this gap, we conducted the integrated analysis based on the bulk sequencing data and scRNA‐seq data to explore the role of immune microenvironment heterogeneity in OSCC and develop the prognostic model to facilitate precise therapy. First, we identified 10 immune cell subtypes in OSCC according to the scRNA‐seq data. In further analysis, we found that cell communication between epithelial cells and tissue stem cells may contributes to the progression of OSCC. Huadong Lai's scRNA‐seq data‐based study also indicated that heterogeneity of epithelium cell is essential for cancer metastasis.^37^ Cancer stem cell is also closely related to invasiveness of cancer.^38^, ^39^ Gunsagar et al.'s scRNA‐seq data analysis demonstrated that cancer stem cell and GULP1 expression contributes to cancer progression and recurrence.^40^ These researches indicated that epithelial cells, tissue stem cells and their interactions are essential for OSCC.

Next, we characterized the immune microenvironment using the bulk sequencing data of OSCC. CD8^+^ T cell infiltration decides the immunotherapy responsiveness of OSCC.^41^, ^42^ Wu et al.'s study demonstrated that interactions between CD8^+^ T cells and Treg cells influence the anti‐tumour immune response and immune evasion of cancer.^43^ Zhou et al. found that tumour antigen presentation‐dependent CD8^+^ T cell proliferation is the core process of OSCC's anti‐tumour process.^44^ Since CD8^+^ T cell plays the essential role in OSCC, we then identified the CD8^+^T cell‐related gene module via WGCNA and chose the module component genes for further analysis.

Single‐cell integration with bulk multi‐omics analysis is critically important in various cancers. Liu et al. developed an immunogenic cell death‐related signature (ICDRS) for clear cell renal cell carcinoma that accurately predicts prognosis, identifies immunotherapy responders.^45^ Wang et al. dissected hepatocellular carcinoma heterogeneity and found that an immunosuppressed subtype characterized by high expression of immunosuppressive genes contributes to an immunosuppressive microenvironment, with BATF and a myeloid‐derived suppressor cell‐like macrophage subtype playing key roles.^46^

Then, we further integrated the result of bulk sequencing and scRNA‐seq data analysis and constructed the gene prognostic model in XGboost. Recent research also demonstrated that model component genes are important immune regulators for OSCC. Franzen, et al. found that methylation of the PDCD1LG2 promoter leads to transcriptional repression of PD‐L1 and PD‐L2 in HNSC.^47^ Research of Lallemant, et al. indicated the diagnostic marker potential of KRT13.^48^

Finally, we validated the model's robustness in validation sets. Immunotherapy has made great progress in the clinical treatment of cancer patients, but its efficacy has been inconsistent. The effectiveness of these treatments varies from patient to patient due to patient heterogeneity. Several studies have proposed biomarkers that predict immunotherapy outcomes, including programmed cell death ligand 1 (PD‐L1) expression and oncogene mutations. However, these tests are invasive, time‐consuming and require large amounts of tumour tissue. The predictive performance of traditional biomarkers is also less satisfactory and unstable. Therefore, there is an urgent need for new biomarkers, especially integrated single‐cell analysis, to effectively predict the outcome of immunotherapy.^49^, ^50^ Results demonstrated that our model has excellent predictive efficiency. Immunotherapy cohort analysis also showed that this model can be potentially applied in evaluating immunotherapy responsiveness of cancer patients. The limitation of our study lies the lack of multi‐omics data enriched with large‐scale clinical information for OSCC, as well as the absence of extensive large‐scale clinical validation for our model.

Previous studies have revealed multiple survival markers in cancers based on computational algorithms. But the biological function of these biomarkers was not demonstrated. To fill this gap, in this study, we performed molecular biological experiments to investigate the regulatory roles of survival markers. Briefly, we examined the expression changes of proliferation and apoptosis markers before and after FCRL4 knock‐down, results showed that inhibition of FCRL4 can suppress cancer cell proliferation and promote cell apoptosis. Additionally, would healing assay and colony formation assays revealed the anti‐cancer role of knockdown FCRL4.

In this study, we integrated single‐cell and bulk‐sequencing data to characterize the immune microenvironment in HNSC. A series of immune signatures were identified by machine learning methods and a prognostic model was constructed subsequently. The prognostic model can effectively predict the prognosis and responsiveness to immunotherapy of OSCC patients, which may facilitate the clinic comprehensive therapy of OSCC.

## AUTHOR CONTRIBUTIONS

**Xingwei Zhang:** Data curation (equal); writing – original draft (equal); writing – review and editing (equal). **Fan Yang:** Data curation (equal); formal analysis (equal); writing – original draft (equal); writing – review and editing (equal). **Chen Dong:** Data curation (equal); writing – original draft (equal); writing – review and editing (equal). **Baojun Li:** Methodology (equal); software (equal). **Shuo Zhang:** Data curation (equal); software (equal). **Xiaohui Jiao:** Conceptualization (equal); project administration (equal); writing – review and editing (equal). **Dong Chen:** Conceptualization (equal); project administration (equal); supervision (equal); writing – review and editing (equal).

## FUNDING INFORMATION

This work was supported by the Research Project of the First Affiliated Hospital of Harbin Medical University (2019M22).

## CONFLICT OF INTEREST STATEMENT

The authors declare no conflicts of interest.

## WRITING DISCLOSURE

No writing assistance was utilized in the production of this manuscript.

## Supporting information


Figure S1.

Figure S2.

Figure S3.

Figure S4.

Table S1.

Table S2.

Data S1.

Data S2.

Data S3.

Data S4.

Data S5.

## Data Availability

Data available on request from the authors.
