# Supplementary material for: Identification and analysis of a cell communication prognostic signature for oral squamous cell carcinoma at bulk and single‐cell levels
Source: J Cell Mol Med. 2024 Nov 24;28(22):e70166. doi: 10.1111/jcmm.70166 (PMC11586053; doi:10.1111/jcmm.70166)
Supplement: Supplementary file 1 — Figure S1. [file JCMM-28-e70166-s005.pdf]

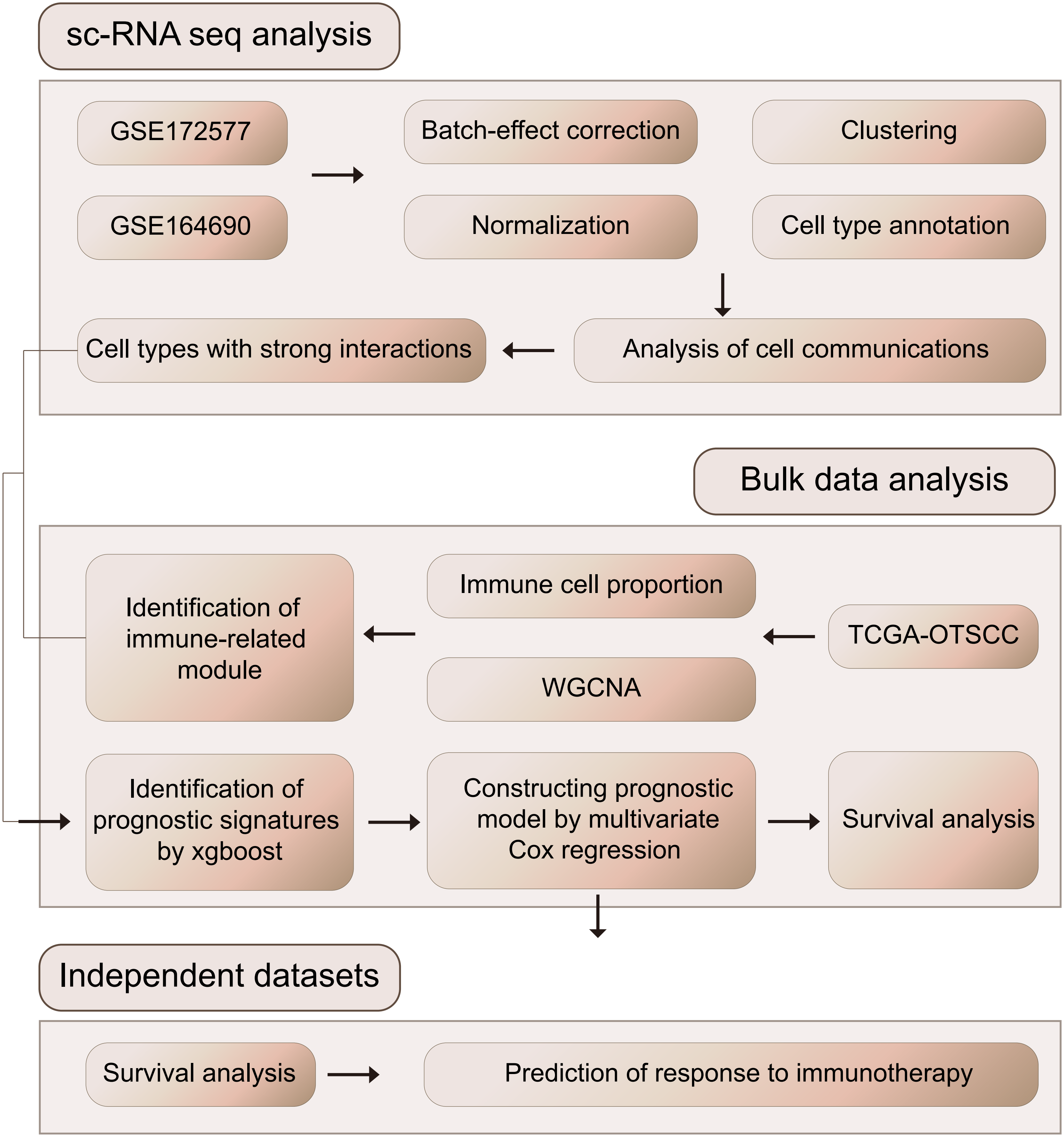

sc-RNA seq analysis
GSE172577
GSE164690
Batch-effect correction
Normalization
Clustering
Cell type annotation
Analysis of cell communications
Cell types with strong interactions
Bulk data analysis
Immune cell proportion
WGCNA
TCGA-OTSCC
Identification of immune-related module
Identification of prognostic signatures by xgboost
Constructing prognostic model by multivariate Cox regression
Survival analysis
Independent datasets
Survival analysis
Prediction of response to immunotherapy
