# Supplementary figures and images for: Identification and analysis of a cell communication prognostic signature for oral squamous cell carcinoma at bulk and single‐cell levels
Source: J Cell Mol Med. 2024 Nov 24;28(22):e70166. doi: 10.1111/jcmm.70166 (PMC11586053; doi:10.1111/jcmm.70166)

A

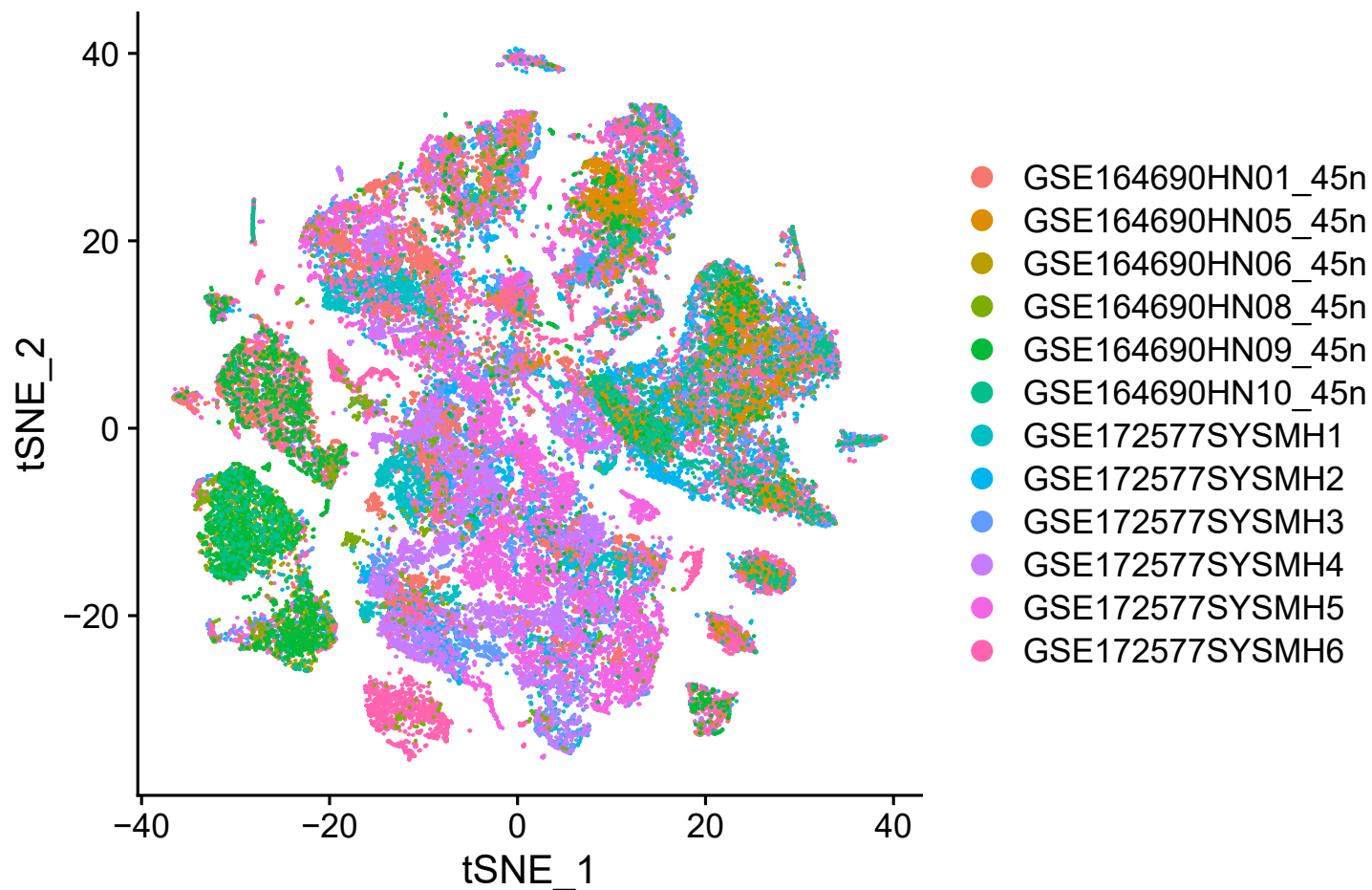

Supplement: Supplementary file 2 — Figure S2. [file JCMM-28-e70166-s003.pdf]

A

Cluster Dendrogram

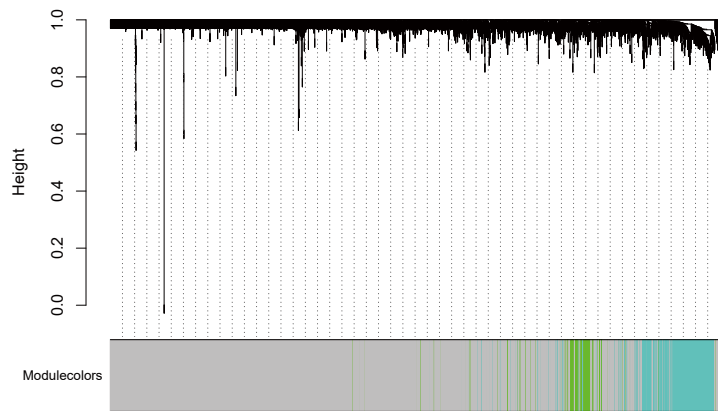

B

Cluster Dendrogram

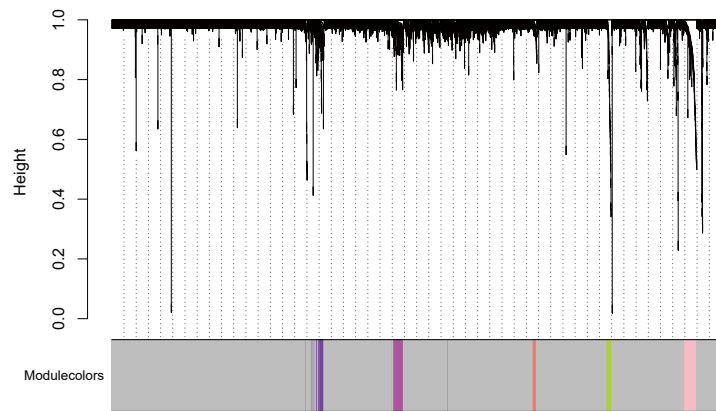

C

Cluster Dendrogram

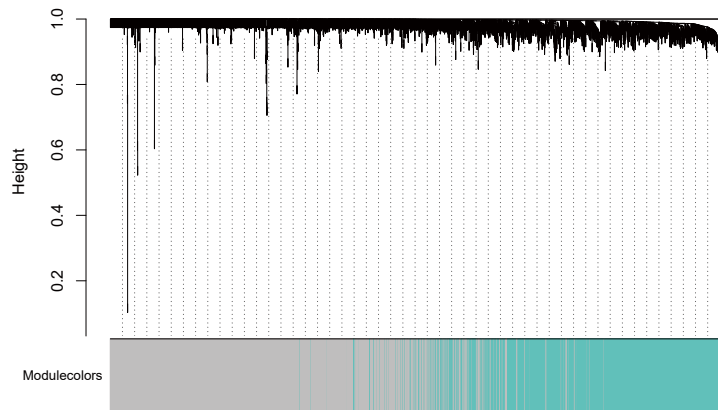

D

Cluster Dendrogram

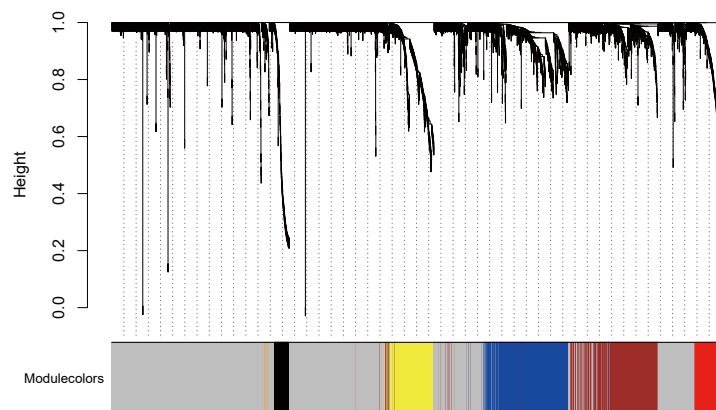

Supplement: Supplementary file 3 — Figure S3. [file JCMM-28-e70166-s010.pdf]

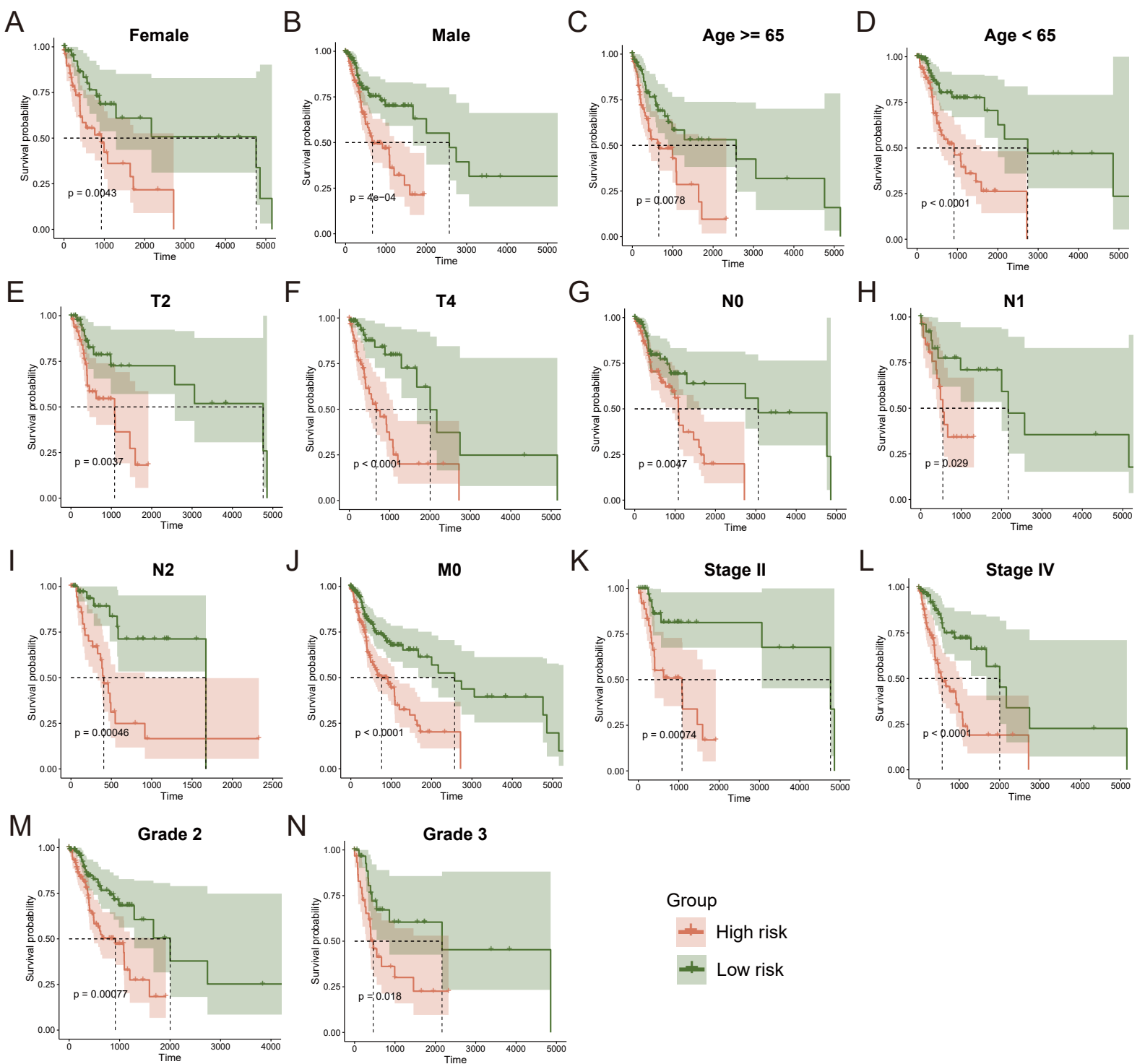

Supplement: Supplementary file 4 — Figure S4. [file JCMM-28-e70166-s011.pdf]
