# Supplementary material for: Identification and analysis of a cell communication prognostic signature for oral squamous cell carcinoma at bulk and single‐cell levels
Source: J Cell Mol Med. 2024 Nov 24;28(22):e70166. doi: 10.1111/jcmm.70166 (PMC11586053; doi:10.1111/jcmm.70166)
Supplement: Supplementary file 5 — Table S1. [file JCMM-28-e70166-s008.docx]

Table S1: siRNA sequences

| Gene | Guide | Passenger |
| --- | --- | --- |
| FCRL4#1 | AACUGAAAUCCAUUGCAAGUC | CUUGCAAUGGAUUUCAGUUCU |
| FCRL4#2 | AUAGAACUGAAAUCCAUUGCA | CAAUGGAUUUCAGUUCUAUGC |
