# Supplementary material for: Identification and analysis of a cell communication prognostic signature for oral squamous cell carcinoma at bulk and single‐cell levels
Source: J Cell Mol Med. 2024 Nov 24;28(22):e70166. doi: 10.1111/jcmm.70166 (PMC11586053; doi:10.1111/jcmm.70166)
Supplement: Supplementary file 6 — Table S2. [file JCMM-28-e70166-s009.docx]

Table S2: Primer sequences

| Gene | Forward | Reverse |
| --- | --- | --- |
| TP53 | GCCCATCCTCACCATCATCACAC | GCACAAACACGCACCTCAAAGC |
| CASPASE3 | GCTGAGCTGCCTGTAACTTGAGAG | GCGTATGGAGAAATGGGCTGTAGG |
| PCNA | GCGTAGCAGAGTGGTCGTTGTC | AGGCGGGAAGGAGGAAAGTCTAG |
| FCRL4 | GGGTAACATCCACAAGCACAGTCC | CTTCAGCCACGGAGCAGACAAG |
| GAPDH | GAAGGTGAAGGTCGGAGTC | GAAGATGGTGATGGGATTTC |
